# Regional Brain Activity Alterations in Social Anxiety Disorder Revealed by Seed‐Based d Mapping With Permutation of Subject Images

**DOI:** 10.1002/brb3.71469

**Published:** 2026-05-05

**Authors:** Sen Li, Ziqi Zhang, Shanling Ji, Aoxue Zhang, Shengbo Han, Zhengyang Chang, Kun Li, Jian Cui, Hao Yu, Chuanxin Liu, Cong Zhou

**Affiliations:** ^1^ School of Mental Health Jining Medical University Jining China; ^2^ School of Clinical Medicine Jining Medical University Jining China; ^3^ College of Integrated Traditional Chinese and Western Medicine Jining Medical University Jining China; ^4^ Department of Psychiatry Shandong Daizhuang Hospital Jining China; ^5^ Department of Psychology Affiliated Hospital of Jining Medical University Jining China

**Keywords:** functional magnetic resonance imaging, neuroimaging, seed‐based d mapping, social anxiety disorder

## Abstract

**Introduction**: Social anxiety disorder (SAD) is a commonly occurring mental health condition characterized by excessive fear and anxiety in social situations. The disorder significantly impacts individuals' daily functioning and is often associated with a range of emotional and physiological symptoms. Understanding the neural basis of SAD is crucial for developing effective treatments.

**Methods**: This meta‐analysis utilized anisotropic effect‐size seed‐based d mapping with permutation of subject images (SDM‐PSI) to examine brain activity alterations in SAD patients. We systematically reviewed neuroimaging studies, focusing on resting‐state functional magnetic resonance imaging (fMRI) data, and analyzed the reported regional brain activity alterations. Our search encompassed studies published up to July 31, 2024, and applied strict inclusion and exclusion criteria to ensure the reliability of the findings.

**Results**: The analysis revealed increased functional activity in the left superior parietal gyrus, right cerebellum, and right supramarginal gyrus, along with decreased activity in the left insula and right supplementary motor area in SAD patients compared to healthy controls (HC). Meta‐regression analysis indicated a negative correlation between left insula activity and age, and between right supplementary motor area activity and symptom severity.

**Conclusion**: The findings provide evidence for distinct neural signatures in SAD, emphasizing the pivotal role of key brain regions in the disorder's pathophysiology. These results contribute to the understanding of the neural correlates of SAD and may guide the development of therapeutic strategies in the future.

## Graphical abstract

This SDM‐PSI meta‐analysis identified consistent regional brain activity alterations in social anxiety disorder, including hyperactivity in the left superior parietal gyrus, right cerebellum, and right supramarginal gyrus, and hypoactivity in the left insula and right supplementary motor area, with age and symptom severity modulating specific regions.

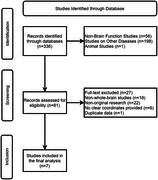

## 1 Introduction

Social anxiety disorder (SAD) is a common and debilitating mental disorder (Carlton et al. 2023), primarily characterized by a persistent fear in one or more social situations, which may stem from past experiences, family background, upbringing, and other factors (Leichsenring et al. 2017; Stein and Stein 2008). Patients with SAD often exhibit an unnecessary fear of social situations, leading to withdrawal or avoidance of social behavior, which can impact their ability to study, work, and engage in social activities (Bögels et al. 2010). Symptoms of SAD are divided into two major categories: physical symptoms and psychological symptoms. Physical symptoms include rapid heartbeat, blushing, trembling, nausea, and diarrhea; in severe cases, it can progress to panic attacks. Psychological symptoms include anxiety and fear. Severe cases of SAD may also be comorbid with depression, obsessive‐compulsive disorder, and other conditions (Adams et al. 2019; Leichsenring et al. 2017). In recent years, the prevalence of SAD has been on the rise, particularly during adolescence, a peak period for the disorder. Globally, an estimated 8.4%–15% of individuals are diagnosed with SAD (Heimberg et al. 2014). Despite its high prevalence, SAD often goes underdiagnosed or misdiagnosed (Maron et al. 2018). SAD is typically a chronic and elusive disorder with a low likelihood of spontaneous remission (Keller 2003; Leichsenring et al. 2017), thereby placing a significant financial burden on patients and their families (Maron et al. 2018). Consequently, timely and effective diagnosis and intervention are imperative for managing the disorder, facilitating rehabilitation, preventing comorbid conditions, and alleviating the socioeconomic impact of SAD (Lecrubier 1998).

Functional magnetic resonance imaging (fMRI) is an emerging neuroimaging technology (Fox and Raichle 2007) that creates images by measuring changes in the magnetic resonance signal intensity based on the oxygen saturation of brain tissue and the hemodynamic response (Logothetis et al. 2002). It is primarily used to study the functional characteristics of the brain or spinal cord in humans and animals and has advantages such as flexibility, safety, noninvasiveness, and strong repeatability (Huettel 2012; Greicius et al. 2003). fMRI has also played an important role in exploring the neuropathological mechanisms of SAD. In the study of SAD, fMRI provides a means to gain a deeper understanding of brain function changes, thus better comprehending the pathological mechanisms of SAD. By observing the brain's response in SAD patients during social situations, fMRI can identify the brain regions activated under normal social conditions and those suppressed in SAD patients. This technology helps to deepen the understanding of the neuropathological mechanisms of SAD, providing theoretical support for the development of more effective and targeted treatments (Zhu et al. 2017).

So far, there has been a substantial amount of neuroimaging research on SAD, revealing complex and widespread brain function alterations in SAD patients. These changes involve multiple brain regions and provide significant evidence for understanding the neuropathological basis of SAD. However, the research findings exhibit a degree of inconsistency. Functional abnormalities in specific brain regions may lead to changes in emotional regulation, adaptive capacity, and attentional regulation in SAD patients (Carlton et al. 2023; Torrisi et al. 2019). Recent investigations reveal that, in contrast to healthy controls (HC), individuals with SAD exhibit enhanced resting‐state functional connectivity between the central nucleus of the amygdala and the lateral orbitofrontal cortex and superior temporal sulcus, while functional connectivity between the striatum and the dorsolateral prefrontal cortex (DLPFC) and occipital cortex is reduced (Torrisi et al. 2019). Task‐based fMRI studies have found (Lucherini Angeletti et al. 2021) that during symptom‐sensitive tasks, SAD patients exhibit increased activity in the default mode network (DMN), amygdala (AMG), and salience network (SN) regions. Research by Y. Zhang, Zhu, et al. 2015) revealed that compared to HC, SAD patients show lower amplitude of low‐frequency fluctuations (ALFF) in the DLPFC, medial prefrontal cortex (MPFC), superior temporal gyrus, and insula, while the ALFF in the middle occipital gyrus is higher. In addition, other studies have shown that, compared to HC, SAD patients have decreased local coherence (ReHo) within the bilateral angular gyrus and left MPFC within the DMN, suggesting impaired perception of socially relevant emotional states and self‐referential mental representations. Moreover, there are functional abnormalities within the right DLPFC and right inferior parietal lobule within the central executive network (CEN), reflecting cognitive control deficits related to social anxiety. ReHo in the left middle occipital gyrus significantly increases, aligning with the heightened vigilance and expressiveness in social interactions observed in patients during the resting state (Qiu et al. 2011).

Seed‐based d mapping with permutation of subject images (SDM‐PSI) is an advanced neuroimaging meta‐analysis technique that allows for the re‐computation of local imaging changes based on the coordinates reported in previous studies (Wu et al. 2022). Given the significant variability in the existing neuroimaging research results on SAD, this study aims to conduct a coordinate‐based meta‐analysis of the fMRI studies on brain function alterations in SAD patients. The goal is to identify the most significant and consistent brain function changes, thereby establishing a robust theoretical foundation for the identification and intervention of SAD in psychological therapy. Several systematic reviews and meta‐analyses have synthesized the growing body of fMRI research in SAD, providing preliminary but important insights into the neural basis of this disorder. Task‐based fMRI meta‐analyses have consistently identified altered activation in limbic and prefrontal regions. For example, Yu et al. (2021) conducted an activation likelihood estimation (ALE) meta‐analysis of 37 task‐based fMRI studies and reported that SAD patients showed significantly lower activation of the left anterior cingulate gyrus compared to HC. Their subgroup analysis further revealed that during emotional face processing, SAD patients exhibited significantly higher activation in the right supramarginal gyrus and angular gyrus. Gentili et al. (2015) performed a meta‐analysis of 23 fMRI studies on face perception in SAD and identified significant clusters of hyperactivation in bilateral amygdala, globus pallidus, superior temporal sulcus, visual cortex, and prefrontal cortex in response to faces. However, these task‐based findings cannot directly inform our understanding of resting‐state brain function, which reflects intrinsic neural activity in the absence of external tasks and may capture different aspects of SAD pathophysiology. For resting‐state fMRI specifically, a comprehensive systematic review by Mizzi et al. (2021) synthesized findings from 35 studies (795 SAD patients, 816 controls) and concluded that individuals with SAD display both higher and lower connectivity between frontal‐amygdala and frontal‐parietal regions, with frontal regions being the most consistently implicated across analysis methods. Notably, this review highlighted that small sample sizes and variation in analytical approaches have contributed to inconsistent findings, and explicitly called for more systematic synthesis of resting‐state evidence. Kim and Yoon (2018) reviewed resting‐state functional connectivity studies in anxiety disorders and reported that increased connectivity within the salience network is a consistent finding in SAD. Despite these valuable contributions, no existing meta‐analysis has specifically targeted resting‐state local brain activity (ReHo and ALFF/fALFF) in SAD using the advanced SDM‐PSI method, which offers methodological advantages including permutation‐based inference and the ability to conduct meta‐regression analyses examining clinical and demographic moderators. Therefore, while the literature provides abundant preliminary evidence that SAD is associated with brain function alterations, the precise nature and consistency of these alterations—particularly in the resting state—remain unresolved. The present meta‐analysis directly addresses this gap by providing the first systematic, quantitative synthesis of resting‐state local activity studies in SAD.

## 2 Methods

### 2.1 Selection of Studies

We conducted a search in the Web of Science, PubMed, and China National Knowledge Infrastructure (CNKI) databases for peer‐reviewed papers published up until July 31, 2024. The search terms used were: (“social phobia” or “social anxiety” or “socially anxious”) and ([“resting state” or “resting‐state” or “at rest” or “resting”] or [“amplitude of low frequency fluctuation” or “fractional amplitude of low frequency fluctuation” or “ALFF” or “fALFF”] or [“regional homogeneity” or “ReHo” or “local connectivity” or “coherence”]).

### 2.2 Study Selection Criteria

For inclusion and exclusion criteria: Inclusion Criteria: (1) original research on resting‐state fMRI in SAD; (2) studies that compare the differences in local brain function between SAD patients and HC; (3) the results must be presented using Montreal Neurological Institute (MNI) coordinates or Talairach coordinates; (4) the thresholds used in the studies must be corrected or controlled for range. Exclusion criteria: (1) non‐original studies, such as case reports, conference proceedings, reviews, meta‐analysis reviews, and so on; (2) studies not focused on humans; (3) studies that do not compare brain function between SAD patients and HC; (4) studies that only analyze specific regions of interest (ROI); (5) studies that do not provide clear coordinates, coordinate systems (e.g., Talairach coordinates or MNI coordinates), or specific information about the participants (e.g., age, gender, disease duration); (6) studies that present results based on duplicate data sets.

### 2.3 Quality Assessment and Data Extraction

The meta‐analysis was conducted following the PRISMA guidelines (Page et al. 2021) and the standards for neuroimaging meta‐analyses (Müller et al. 2018). The following data was extracted: general information; sample characteristics, including sample size, gender, age, and disease duration; and imaging data, including peak activation coordinates and *t*‐values, as well as statistical thresholds.

### 2.4 Statistical Analysis

Meta‐analyses were performed using the SDM‐PSI software (version 5.15, http://www.sdmproject.com/). Peak coordinates and their corresponding effect sizes (*t*‐values) were extracted from each included dataset and prepared into text files following the standard naming conventions required by the software. An SDM table was then generated to organize the extracted data. The analysis strictly followed the standard workflow of the SDM‐PSI protocol, which consists of global analysis, preprocessing, mean analysis, threshold analysis, and mask creation. The software reconstructs effect size and variance maps of the difference between SAD patients and HCs using the extracted peak coordinates and their associated statistics, then synthesizes these maps within a random‐effects model. In this framework, merging across studies is not a simple average but rather employs an inverse variance weighting strategy, where the weight assigned to each study is inversely proportional to the variance of its effect estimate. This approach integrates sample size information and reduces the influence of low‐precision or high‐variance studies, thereby minimizing the potential exaggeration effects arising from multiple comparisons or multi‐peak reports. Heterogeneity evaluation is embedded within the algorithm. The analyses were conducted using the “Functional MRI or PET” template within the software.

We used the default threshold of voxel‐level *p* < 0.005 with a cluster extent of 10 voxels and peak *Z* > 1, uncorrected. These threshold parameters follow the recommendations of the developers of SDM‐PSI (Radua et al. 2012), who found that an uncorrected threshold of *p* < 0.005 approximates a corrected *p* < 0.05 and optimally balances sensitivity and specificity. Given that factors such as the participants' age and disease duration may have potential effects on brain function, meta‐regression analysis based on the Monte Carlo random algorithm was employed to further explore the roles of these variables. Following the recommendations of the SDM‐PSI software developers, the threshold for regression analysis was set to *p* = 0.0005 to ensure the robustness of the results. The scope of the regression analysis was limited to brain regions with detected differences in the primary meta‐analysis, focusing on the potential influencing factors in these specific regions.

### 2.5 Jackknife Sensitivity, Heterogeneity, and Publication Bias Analyses

To evaluate the robustness of the findings, we performed a whole‑brain jackknife sensitivity analysis. This iterative analysis eliminates one dataset at a time to determine the reliability of the results; if all or most combinations yield significant results for a given region, the findings are considered highly reproducible. For each significant cluster from the patient‑control comparison, the *I* ^2^ statistic was used to assess heterogeneity, with *I* ^2^ > 50% considered indicative of significant heterogeneity (Rücker et al. 2008). Publication bias was assessed using Egger's test to evaluate asymmetry of funnel plots for each significant cluster, with *p* < 0.05 regarded as indicating significant publication bias (Egger et al. 1997).

The authors confirm that no artificial intelligence tools, including large language models, were used in the generation of any part of this manuscript.

## 3 Results

### 3.1 Literature Search Results

**FIGURE 1:**
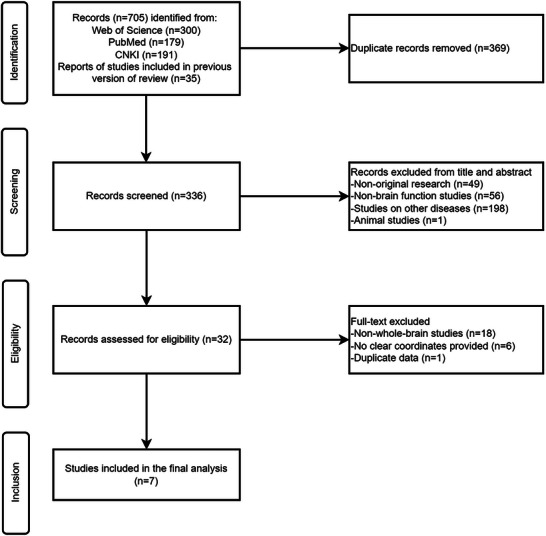
Flowchart of literature search.

A total of 336 articles were initially identified, with a final selection of 7 articles encompassing 7 datasets (He et al. 2024; Qiu et al. 2015; Qiu et al. 2011; C. Yuan, Zhu, Ren, et al. 2018; M. Yuan et al. 2016; M. Yuan, Zhu, Qiu, et al. 2018; W. Zhang, Yang, et al. 2015). Among them, the studies by Qiu et al. (2011) and Qiu et al. (2015) used partially overlapping subject cohorts but employed different analytical metrics (ReHo vs. ALFF). To assess whether this overlap influenced our results, we conducted exclusion and jackknife sensitivity analyses (see Sections 2.5 and 3.4), which confirmed that the overlapping samples did not drive the primary findings. These studies collectively included 194 SAD patients and 241 HC, encompassing 92 coordinates. The basic characteristics of these articles are detailed in Table 1, and the inclusion and exclusion process of the literature is visually presented in Figure 1.

**TABLE 1:** Basic characteristics of the seven studies included in the analysis.

Included studies	Sample size (number of female cases)	Mean age (years)	Duration of illness (years)	Severity	Medication status	Comorbidities in patient group	Analysis method
	SAD HC	SAD HC					
Qiu et al. (2011)	20(6) 20(6)	22.9 21.7	3.8	LSAS: 53.9 HAMD: 7.5 HAMA: 7.5 STAI: 48.3	Naive	None	ReHo
Qiu et al. (2015)	20(6) 19(5)	22.9 21.4	3.8	LSAS: 53.9 HAMD: 7.5 HAMA: 7.5 STAI: 48.3	Naive	None	ALFF
W. Zhang et al. (2015)	40(14) 40(14)	26.0 24.8	7.8	LSAS: 65.4	Anxiolytics (12)	None	ReHo
M. Yuan et al. (2016)	15(4) 18(5)	27.0 26.3	NA	LSAS: 64.9 HAMD: 9.0 HAMA: 11.9	Naive	None	ReHo
C. Yuan et al. (2018)	43(16) 43(17)	29.0 30.1	NA	LSAS:69.2 HAMD: 12.5 HAMA: 14.1	Naive	Depression (10)	ALFF
M. Yuan et al. (2018)	15(5) 19(6)	27.0 26.0	9.1	LSAS: 78.9 HAMD:11.0 HAMA: 14.5	Antidepressants (4)	None	ALFF
He et al. (2024)	41(16) 82(31)	26.2 30.4	NA	LSAS: 56.9 BDI: 27.1 PHQ‐9: 13.5	Antipsychotics (13) Antidepressants (11) Anxiolytic (12) Hypnotics/Sedatives (9) Antiparkinsonian (2) Others (6) Unmedicated (18)	None (22) MDD (8) GAD (2) MDD + GAD (1) MDD + GAD + Agoraphobia (1) GAD + agoraphobia + dysthymia (1) Agoraphobia (2) Agoraphobia + PD (3) Dysthymia (1)	ALFF

Abbreviations: ALFF, amplitude of low‐frequency fluctuation; HAMA, Hamilton Anxiety Rating Scale; HAMD, Hamilton Depression Rating Scale; HC, healthy control; LSAS, Liebowitz Social Anxiety Scale; Naïve, medication‐naive (or not Medicated); None, no other diseases; ReHo, regional homogeneity; STAI, State‐Trait Anxiety Inventory.

### 3.2 Meta‐Analysis Results

**FIGURE 2:**
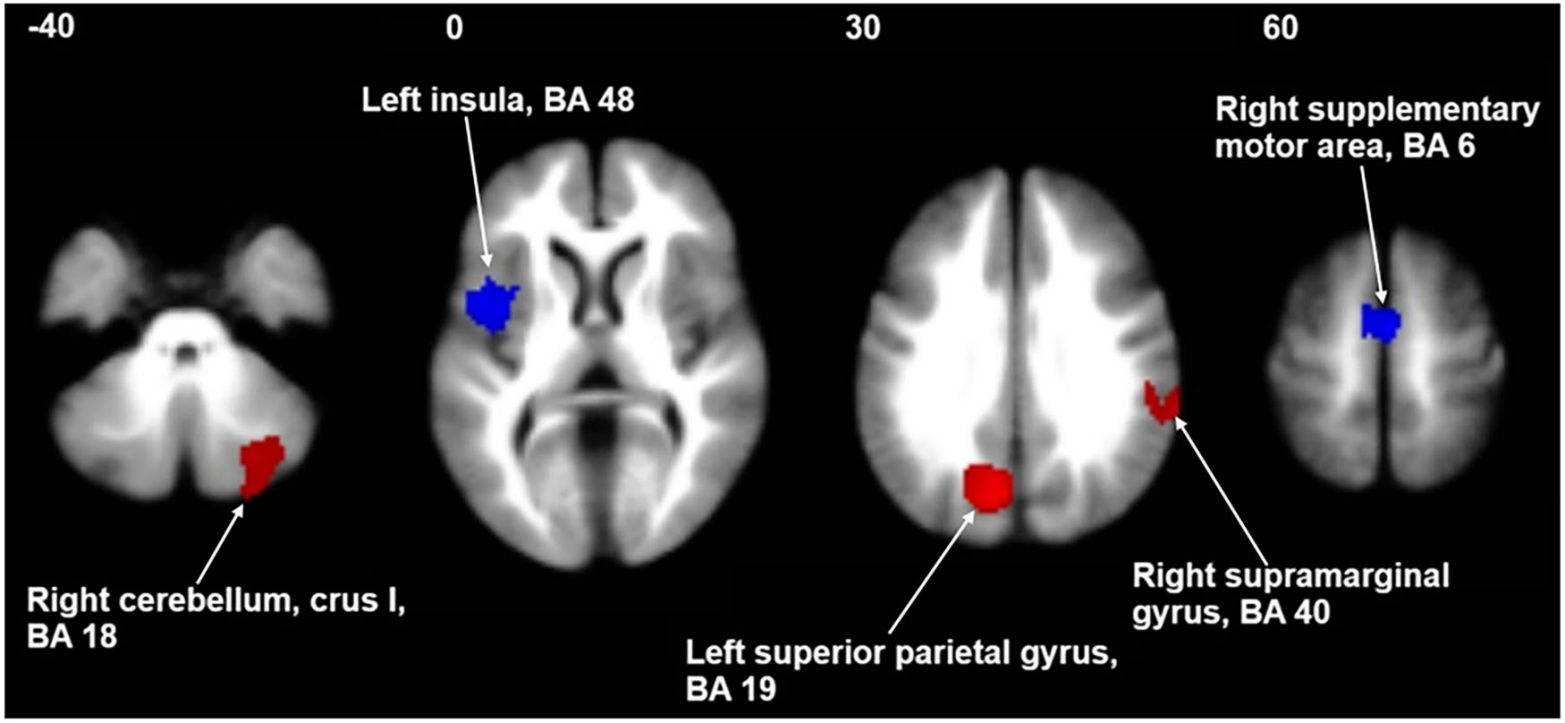
Brain regions with changes in SAD patients compared to healthy controls. Significant clusters are overlaid on MRIcron template in axial views for display purposes only.

The meta‐analysis of brain function in SAD patients reveals abnormally elevated functional activity in the left superior parietal gyrus, right cerebellum, and right supramarginal gyrus regions in SAD patients. In addition, a reduction in functional activity is observed in the left insula and right supplementary motor area (SMA) (Table 2 and Figure 2).

**TABLE 2:** Brain regions showing changes in social anxiety disorder (SAD) patients compared to HC.

Brain region		Peak value		Voxel size	Jackknife sensitivity analysis
	MNI coordinates	SDM *Z* value	*p* value		
**Brain regions with increased function**					
Left superior parietal gyrus, BA 19	−14, −70, 40	2.254	∼0	811	7/7
Right cerebellum, crus I, BA 18	18, −80, −24	1.723	0.001073420	778	6/7
Right supramarginal gyrus, BA 40	64, −38, 36	1.884	0.000227094	219	7/7
**Brain regions with decreased function**					
Left insula, BA 48	−42, 2, 6	−1.941	0.000758648	555	6/7
Right supplementary motor area, BA 6	4, −6, 60	−1.939	0.000768960	339	7/7

Abbreviations: BA, Brodmann area; HC: healthy controls; MNI, Montreal Neurological Institute; SDM, Seed‐based d Mapping.

### 3.3 Meta‐Regression Analysis Results

The meta‐regression analysis demonstrates that in SAD patients, abnormal brain function in the left insula is negatively correlated with the mean age, while abnormal brain function in the right SMA is negatively correlated with the severity of clinical symptoms (Table 3).

**TABLE 3:** Correlation between brain activity changes and clinical characteristics in social anxiety disorder (SAD) patients revealed by meta‐regression analyses.

		MNI coordinates					
Factor	Anatomic label	*X*	*Y*	*Z*	SDM value	*p*	Number of voxels**
Age	Left insula, BA 48	−36	−10	6	−3.497	0.000144482	119
LSAS	Right supplementary motor area, BA 6	2	−10	62	−2.044	0.000180602	79

Abbreviations: BA, Brodmann area; LSAS, Liebowitz Social Anxiety Scale; MNI, Montreal Neurological Institute; SDM, Seed‐based d Mapping. **: *p* < 0.005

### 3.4 Jackknife Sensitivity, Heterogeneity, and Publication Bias Results

The jackknife sensitivity analysis revealed that all significant clusters remained detectable in at least six out of seven jackknife iterations, indicating good reproducibility. Specifically, the left superior parietal gyrus, right supramarginal gyrus, and right SMA were significant in all seven iterations (7/7), while the right cerebellum and left insula were significant in six out of seven iterations (6/7), see Table 2. Heterogeneity analysis showed no significant inconsistency across studies for any of the identified clusters (all *I* ^2^ < 50%). Egger's tests and funnel plots likewise indicated no significant publication bias (all *p* > 0.05). Detailed results are provided in Table S1 and Figure S1.

## 4 Discussion

The current meta‐analysis of fMRI studies on SAD provides a robust examination of the neural correlates associated with this prevalent mental health condition. Our findings reveal significant alterations in brain activity, particularly within the left superior parietal gyrus, right cerebellum, and right supramarginal gyrus, which exhibit increased functional activity, and the left insula and right SMA, which show decreased activity. These neural aberrations are pivotal for understanding the relationship between brain activity alterations and clinical manifestations of SAD, and they suggest potential avenues for future research and therapeutic interventions.

Our findings should be considered in the context of existing reviews and meta‐analyses in the field. Consistent with task‐based meta‐analyses that have implicated the insula and amygdala in SAD (Gentili et al. 2015; Yu et al. 2021), we observed decreased activity in the left insula, a region critically involved in interoceptive awareness and emotional experience. The right supramarginal gyrus hyperactivation identified in our study aligns with the findings of Yu et al. (2021), who reported increased activation in the right supramarginal gyrus and angular gyrus during emotional face processing in SAD, suggesting that this region may be involved in both task‐evoked and resting‐state abnormalities. Furthermore, our identification of cerebellar alterations extends previous network‐based models that have primarily focused on limbic and prefrontal circuits (Kim and Yoon 2018), highlighting the potential role of the cerebellum in the cognitive and affective dimensions of SAD. Importantly, our meta‐analysis extends prior systematic reviews (Mizzi et al. 2021) by providing the first quantitative synthesis of resting‐state local brain activity in SAD, thereby moving beyond qualitative descriptions of heterogeneity to statistically identify spatially convergent abnormalities. The meta‐regression findings linking insular dysfunction to age and SMA dysfunction to symptom severity offer novel insights that were not possible in previous narrative syntheses, underscoring the value of quantitative meta‐analytic approaches in advancing our understanding of SAD neurobiology.

The superior parietal gyrus, a region critical for sensory integration and spatial awareness (Sun et al. 2022), exhibits heightened activity in SAD patients. This increased activity may reflect an altered processing of social cues and an exaggerated self‐consciousness during social interactions, which is a hallmark of SAD (Clark and McManus 2002). The superior parietal gyrus is known to play a crucial role in directing attention to relevant stimuli and is implicated in the multisensory integration necessary for social perception (Greene and Soto 2014). In the context of SAD, this heightened activity could signify an overemphasis on sensory details that are misconstrued as threatening or judgmental, leading to heightened anxiety and discomfort in social situations. The right cerebellum, increasingly recognized for its role in cognitive and affective processing (Rudolph et al. 2023), also shows increased activity in SAD patients. This may reflect disrupted cerebellar modulation of emotional responses, potentially contributing to misaligned temporal coordination of emotional and cognitive processes during social interactions (Mannarelli et al. 2023). In SAD, the heightened activity of the right cerebellum might signal a misalignment in the temporal coordination of emotional and cognitive processes, potentially leading to an intensified and prolonged emotional reaction to social cues.

Our study has also identified significant reductions in functional activity within the left insula and the right SMA in individuals with SAD. The left insula is integral to interoceptive awareness and emotional experience, particularly in social and physiological contexts (R. Zhang et al. 2024). This brain region is also implicated in the experience of emotions, particularly those related to social and physiological conditions (Gasquoine 2014; R. Zhang et al. 2024). Reduced activity in this region may impair internal bodily sensation processing, contributing to heightened self‐consciousness and fear of negative evaluation in SAD. This dysfunction may lead to a diminished ability to detect and respond appropriately to physiological cues during social interactions, potentially exacerbating anxiety symptoms. The right SMA is traditionally thought to be associated with the preparation and initiation of movements(Hertrich et al. 2016). It also plays a role in the cognitive aspects of action, such as conflict resolution and the selection of appropriate responses in social contexts (Hertrich et al. 2016; Nachev et al. 2008). The decreased activity in the right SMA observed in our study could reflect a disruption in the cognitive control of action, which may manifest as difficulty in initiating social interactions or an increased likelihood of socially avoidant behavior in individuals with SAD. This could be particularly relevant during situations that require quick, adaptive responses, where the individual with SAD may experience heightened anxiety and a subsequent avoidance of such interactions. Taken together, the heightened activity in the superior parietal gyrus and right cerebellum may be interpreted through the lens of neural compensation, where these regions might be hyperactivating to compensate for deficits in other brain areas, such as the insula or prefrontal cortex, which show reduced activity.

Meta‐regression revealed a significant negative correlation between age and left insula dysfunction in SAD patients, suggesting that insular abnormalities become more pronounced with age. This age‐related deterioration may contribute to the persistence and escalation of SAD symptoms over time, underscoring the importance of early intervention to mitigate functional decline. This study also identified a significant negative correlation between the severity of clinical symptoms and the abnormal brain function in the right SMA among individuals with SAD. This intriguing finding suggests that as the intensity of SAD symptoms increases, there is a corresponding decrease in the functional activity of the right SMA. The SMA, known for its role in the preparation and initiation of actions, may be particularly sensitive to the cognitive and motor demands of social interactions, which are often challenging for those with SAD (Hertrich et al. 2016; Nachev et al. 2008). This negative correlation could imply that the right SMA's function is compromised under the heightened stress and anxiety experienced by individuals with more severe SAD symptoms. The diminished activity in the right SMA might reflect a disruption in the cognitive control of action (Nachev et al. 2008), potentially leading to difficulties in engaging in social interactions or an increased tendency to avoid them. This could be particularly detrimental in situations that require quick and adaptive social responses, where individuals with SAD may feel overwhelmed and consequently withdraw. The connection between SMA dysfunction and symptom severity underscores the intricate relationship between brain function and the clinical expression of SAD. It points towards the potential role of the right SMA as a neural marker for symptom severity in SAD, which could have implications for the development of targeted therapeutic strategies. By understanding how the right SMA's function is associated with symptomatology, clinicians may be better equipped to monitor treatment progress and adjust interventions to address the specific cognitive and motor challenges faced by individuals with SAD.

This study has several limitations. First, the variability in participant demographics, diagnostic criteria, methodologies, imaging techniques, and data preprocessing across studies might affect both the generalizability of the findings and the comparability of results. This inconsistency suggests a pressing need for more standardized research designs and imaging protocols in future investigations to enhance reliability and validity. Second, the reliance on cross‐sectional data limits the ability to establish causal relationships between brain activity and SAD symptoms, emphasizing the importance of longitudinal studies to track changes over time. Third, potential publication bias may skew the results, as studies with non‐significant findings are less likely to be published, which could lead to an overestimation of the observed effects. Fourth, the modest number of included studies (*n* = 7) and total sample size (194 patients) represent a key limitation. This small evidence base not only increases the risk of both false‑positive and false‑negative findings—despite the robustness suggested by our jackknife sensitivity analyses—but also precluded meaningful subgroup analyses to dissect potential differential contributions of specific resting‐state‑fMRI metrics (e.g., ReHo vs. ALFF/fALFF). Consequently, our findings should be considered preliminary, and future meta‑analyses incorporating a larger corpus of primary studies are essential to validate and refine the neural model of SAD proposed here, as well as to enable metric‑specific investigations. Fifth, although this meta‐analysis was conducted following rigorous methodological standards including PRISMA guidelines and predefined inclusion criteria, it was not preregistered prior to initiation. Preregistration is increasingly recognized as an important tool for enhancing transparency and reducing reporting bias, and its absence represents a limitation of the present study. Future meta‐analyses in this field are encouraged to adopt preregistration as a standard practice. Lastly, the use of different scales to assess symptom severity could introduce variability in the interpretation of clinical data, underscoring the importance of a consistent approach to symptom evaluation in SAD research.

## 5 Conclusion

In conclusion, this meta‐analysis contributes to the growing body of knowledge on the neuropathology of SAD, offering a foundation for developing targeted interventions and advancing our understanding of this complex disorder. The identified neural correlates of SAD may serve as potential targets for therapeutic strategies, highlighting the importance of early and effective treatment to improve outcomes and reduce the socioeconomic burden of SAD.

## Author Contributions

**Sen Li**: conceptualization, data curation, investigation, validation, formal analysis, writing – original draft. **Ziqi Zhang**: resources, data curation, formal analysis. **Shanling Ji**: methodology, software, data curation, visualization. **Aoxue Zhang**: data curation, resources. **Shengbo Han**: data curation, resources. **Zhengyang Chang**: data curation, resources. **Kun Li**: data curation, resources. **Jian Cui**: data curation, resources. **Hao Yu**: resources, writing – review and editing. **Chuanxin Liu**: supervision, validation. **Cong Zhou**: conceptualization, funding acquisition, investigation, data curation, writing – review and editing, methodology, project administration.

## Funding

The Medical and Health Science and Technology Development Plan of Shandong Province (202003061210, 202304011343), the Key Research and Development Plan of Jining City (2021YXNS024), the Cultivation Plan of High‐level Scientific Research Projects of Jining Medical University (JYGC2021KJ006), the Ministry of Education's Industry School Cooperation Collaborative Education Project (220900242232529), the National Natural Science Foundation of China (81901358), the Natural Science Foundation of Shandong Province (ZR2019BH001 and ZR2021YQ55), the Young Taishan Scholars of Shandong Province (tsqn201909146), and the Supporting Fund for Teachers’ Research of Jining Medical University (600903001).

## Ethics Statement

The authors have nothing to report.

## Conflicts of Interest

The authors declare no conflicts of interest.

## Supporting information




**Supplementary Material**: brb371469‐sup‐0001‐SuppMat.docx

## Data Availability

The data compiled for this study is available upon reasonable request to the corresponding authors.
