# Supplementary material for: Regional Brain Activity Alterations in Social Anxiety Disorder Revealed by Seed‐Based d Mapping With Permutation of Subject Images
Source: Brain Behav. 2026 May 5;16(5):e71469. doi: 10.1002/brb3.71469 (PMC13144769; doi:10.1002/brb3.71469)

Supplementary Table 1. Publication bias.

| Cluster | bias | *T*-value | df | *p* | Heterogeneity, *I^2^* |
| --- | --- | --- | --- | --- | --- |
| 1 | 0.25 | 0.14 | 5 | 0.893 | 24.97% |
| 2 | -0.15 | 0.07 | 5 | 0.945 | 26.16% |
| 3 | -1.77 | -0.99 | 5 | 0.369 | 18.66% |
| 4 | 0.18 | 0.13 | 5 | 0.902 | 6.91% |
| 5 | 1.55 | 0.94 | 5 | 0.392 | 14.30% |

Supplementary Fig.1. Funnel plots of identified 5 clusters.

Cluster 1


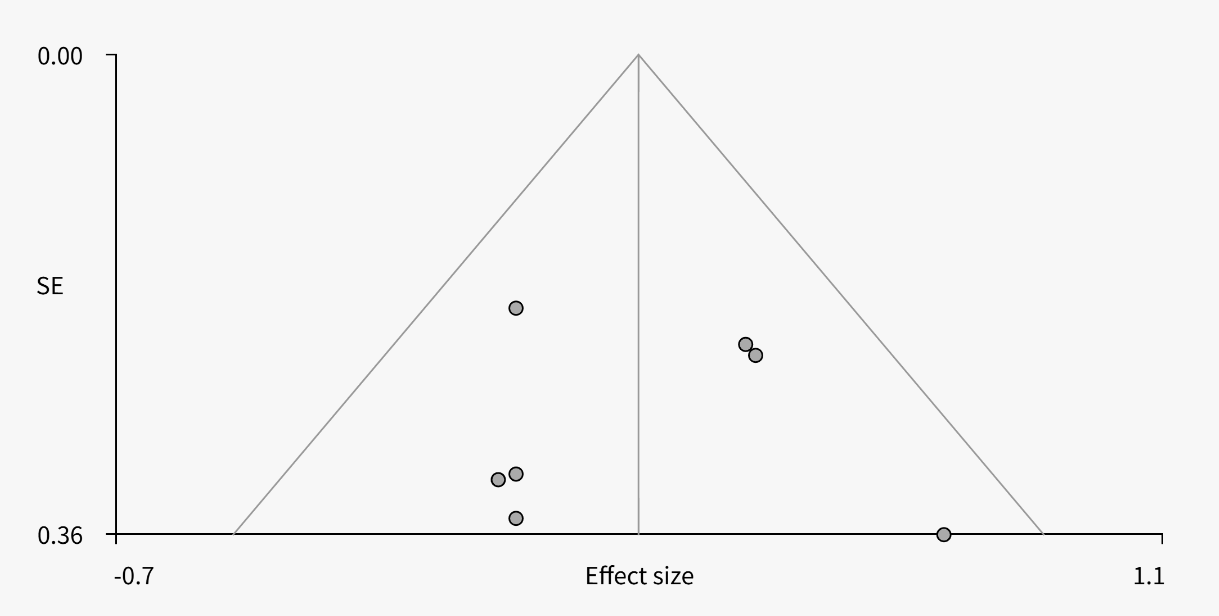


Cluster 2


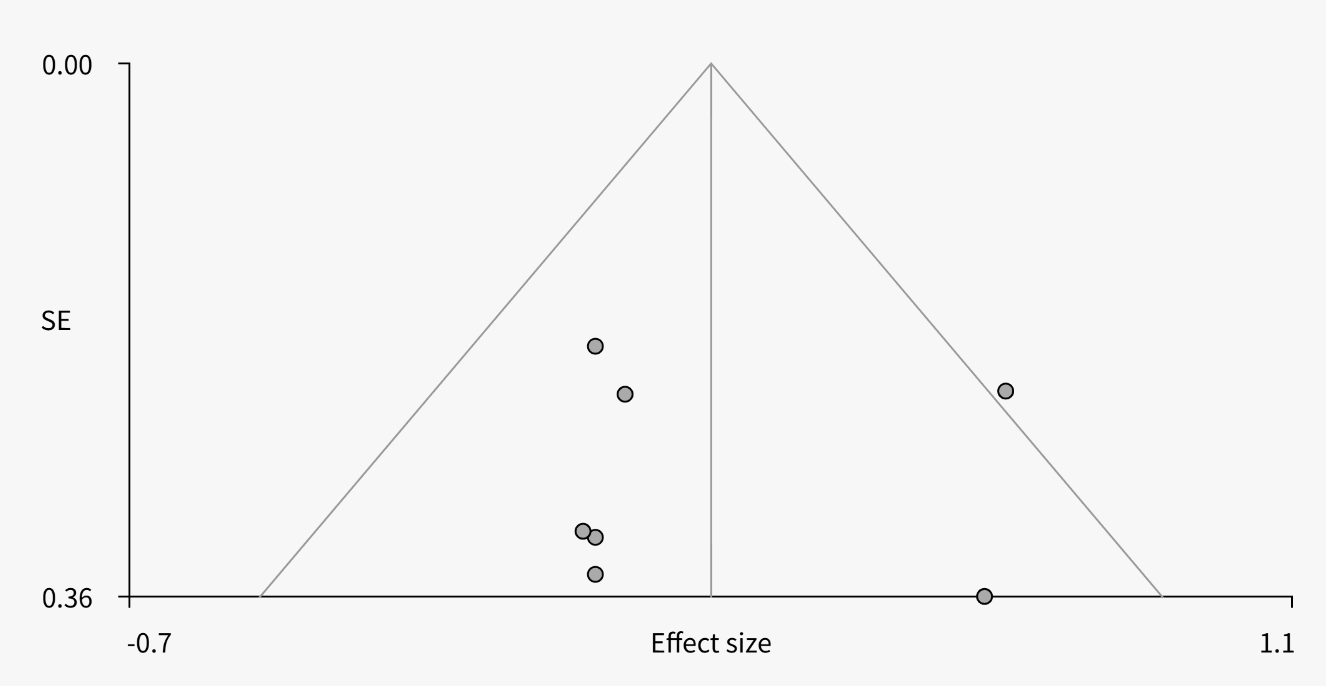


Cluster 3


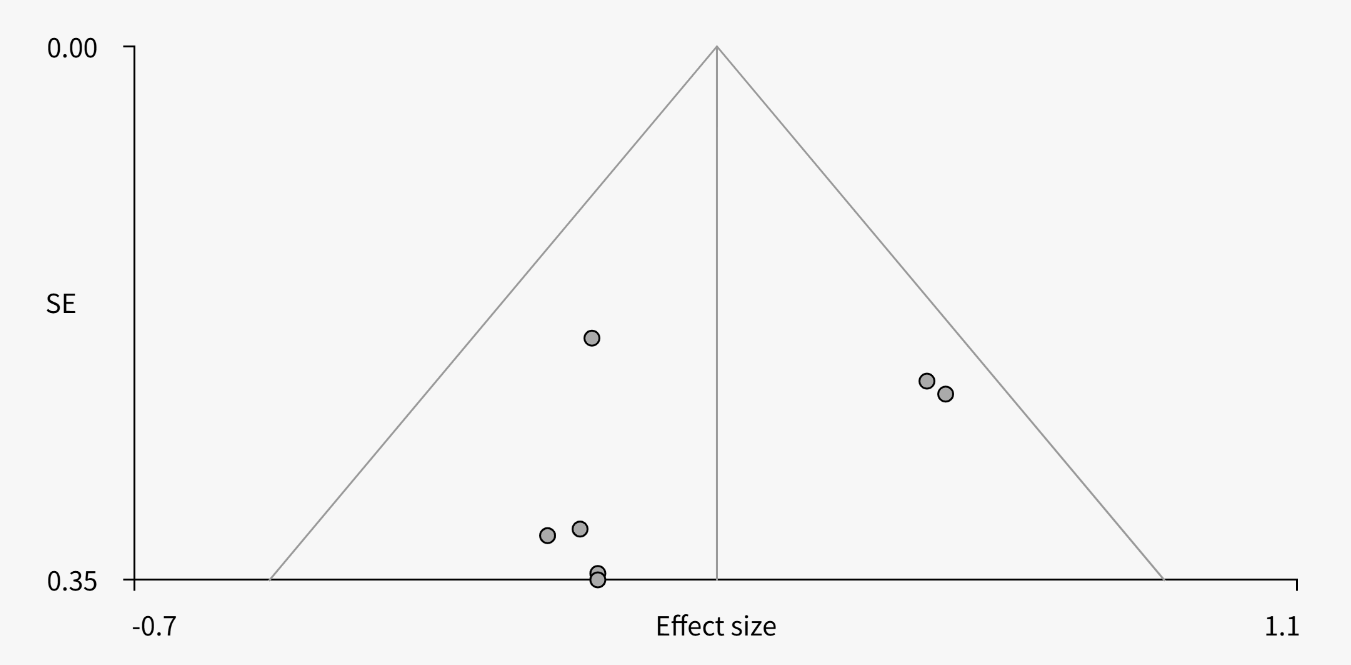


Cluster 4


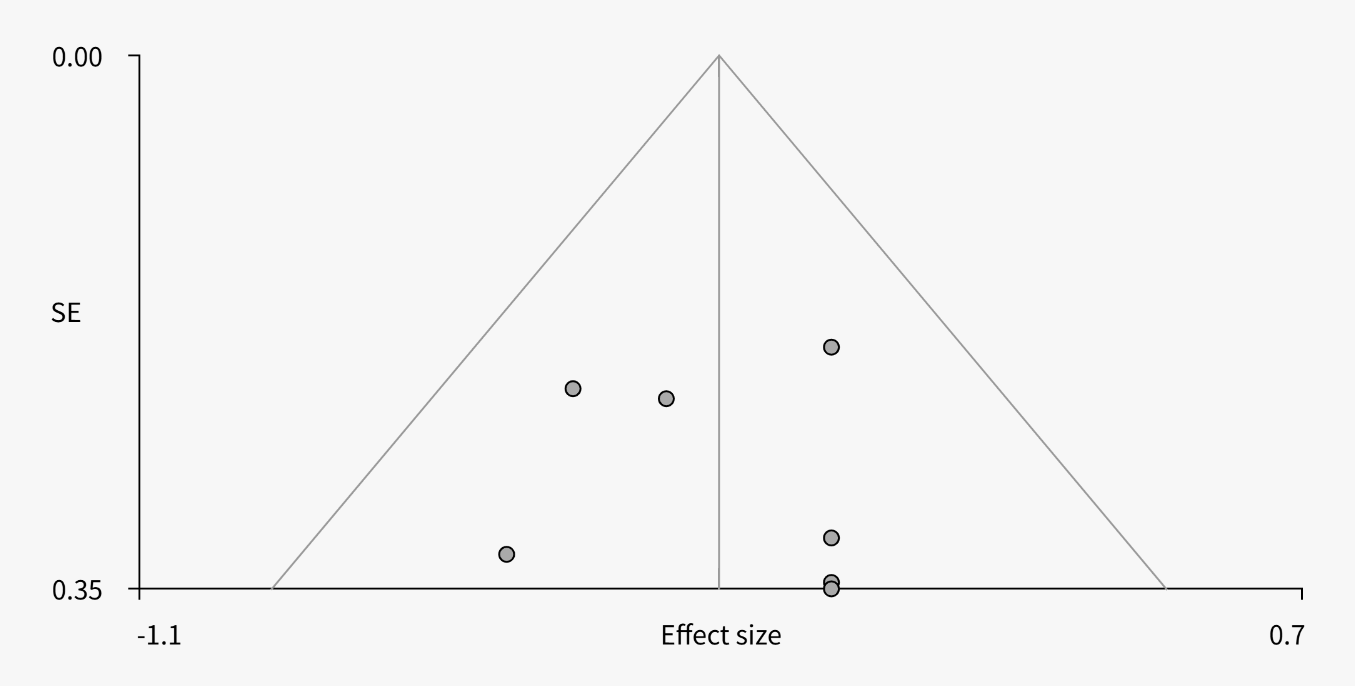


Cluster 5


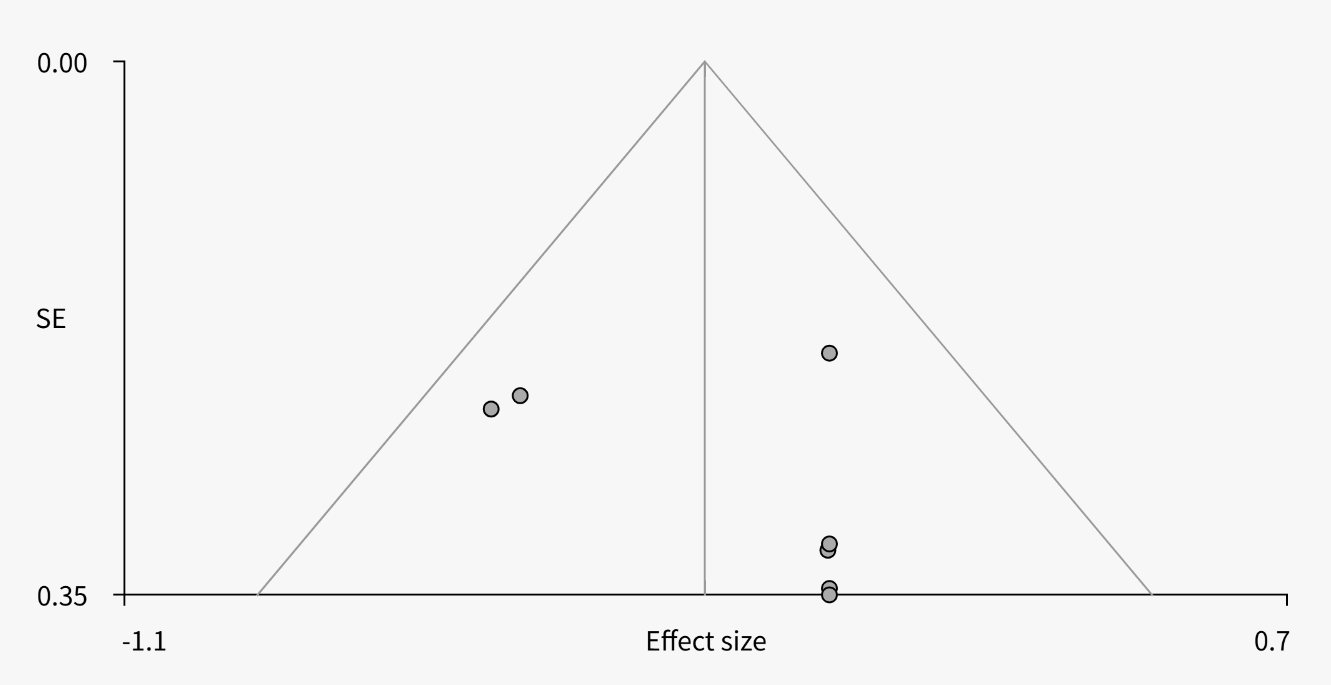

Supplement: Supplementary file 1 — Supplementary Material: brb371469‐sup‐0001‐SuppMat.docx [file BRB3-16-e71469-s001.docx]
